# Prescription-grade crystalline glucosamine sulfate as an add-on therapy to conventional treatments in erosive osteoarthritis of the hand: results from a 6-month observational retrospective study

**DOI:** 10.1007/s40520-022-02151-7

**Published:** 2022-05-30

**Authors:** Sara Tenti, Nicola Veronese, Sara Cheleschi, Iole Seccafico, Olivier Bruyère, Jean-Yves Reginster, Antonella Fioravanti

**Affiliations:** 1 Clinic for the Diagnosis and Management of Hand Osteoarthritis, Rheumatology Unit, Department of Medicine, Surgery and Neuroscience, Azienda Ospedaliera Universitaria Senese, Policlinico Le Scotte, Viale Bracci 1, 53100 Siena, Italy; 2 Geriatric Unit, Department of Internal Medicine and Geriatrics, University of Palermo, Palermo, Italy; 3 Division of Public Health, Epidemiology and Health, Economics, WHO Collaborating Centre for Public Health, Aspects of Musculo-Skeletal Health and Ageing, University of Liege, Liege, Belgium

**Keywords:** Prescription-grade crystalline glucosamine sulfate, Hand osteoarthritis, Erosive hand osteoarthritis, Symptomatic slow-acting drugs for osteoarthritis, Retrospective study, Pain

## Abstract

**Objective:**

To evaluate the efficacy of prescription-grade Crystalline Glucosamine Sulfate (pCGS) as an add-on treatment to conventional therapy, compared to usual therapy alone, in patients with erosive osteoarthritis of the hand (EHOA).

**Methods:**

This 6-month retrospective case–control study included patients with concomitant knee osteoarthritis and symptomatic EHOA. Participants were stratified into two groups based on whether or not pCGS (1500 mg/day) was added to the conventional therapy (education and training in ergonomic principles, exercise and use on-demand of symptomatic drugs) for hand osteoarthritis. Patients were evaluated at baseline, after 3 and 6 months. Primary outcomes were the change from baseline to month 6 in Visual Analogue Scale (VAS) hand pain and in Functional Index for Hand Osteoarthritis (FIHOA) score. A set of secondary parameters was also evaluated.

**Results:**

123 patients were included as follows: 67 treated with pCGS in addition to conventional therapy (pCGS Group) and 56 with conventional therapy alone (Control Group). After 6 months a significant difference in VAS and in FIHOA score (*p* < 0.01 and *p* < 0.001, respectively) was observed in favor of pCGS Group. Similar results were found for morning stiffness duration (*p* < 0.05), health assessment questionnaire (*p* < 0.01) and physical and mental component score of 36-item short form (*p* < 0.05 and *p* < 0.001, respectively). A significant reduction of symptomatic drug consumption at 3 and 6 months was reported in the pCGS Group (*p* < 0.001). No serious adverse event was recorded in both groups.

**Conclusions:**

Despite all the limitations inherent to an observational study, our results suggest the potential effectiveness of pCGS, when used in combination with conventional therapy in EHOA. Further randomized placebo-controlled trials are needed to confirm these positive findings.

**Trial Registration:**

ClinicalTrials.gov, http://www.clinicaltrials.gov, date of registration: February 2, 2022, NCT05237596. The present trial was retrospectively registered.

**Supplementary Information:**

The online version contains supplementary material available at 10.1007/s40520-022-02151-7.

## Introduction

Hand Osteoarthritis (HOA) is a very common condition and one of the main leading causes of disability. The prevalence of symptomatic HOA ranges from 13 to 26% in women older than 70 years with an estimated lifetime risk of 40% [1, 2]. Erosive osteoarthritis (EHOA) is a peculiar variant of HOA, featured by prominent signs of inflammation, high severity progression and typical radiographic changes, as characteristic central erosions with collapse of the subchondral bone and a ‘gull-wing’ or ‘saw-tooth’ deformity [3–6]. It is still highly debated if EHOA represents an advanced stage of the classical HOA or rather a separate entity with inflammatory features [7, 8]. EHOA poses a significant clinical challenge, considering the substantial disability and the negative impact on quality of life (QoL), the paucity of symptomatic effective treatments and the lack of disease modifying anti-rheumatic drugs (DMARDs) [9–20].

Specific guidelines for EHOA management have not yet been provided, so they are actually extrapolated by the 2018 update of the European League against Rheumatism (EULAR) recommendations for the treatment of HOA. These suggest an individualized and multidisciplinary approach, including a combination of non-pharmacological and pharmacological strategies. Among the first ones, education and training in ergonomic principles, use of assistive devices, exercise to improve function and muscle strength and to reduce pain should be offered to every patient [21]. Among the pharmacological options, topical non-steroidal anti-inflammatory drugs (NSAIDs), oral analgesics and intra-articular injection of glucocorticoids in case of painful interphalangeal (IP) joints are recommended [21]. Chondroitin sulfate (CS) is the only symptomatic slow-acting drug for osteoarthritis (SYSADOAs) included in the EULAR recommendations update, considering the randomized controlled trial (RCT) demonstrating its efficacy in relief pain and improving functionality in HOA patients [21, 22]. Also, in EHOA, CS showed some promising results [23]. Finally, EULAR recommends against the use of conventional or biological DMARDs, as hydroxychloroquine, different Tumor Necrosis Factor (TNF)-inhibitors and anti-interleukin (IL)-1 and IL-6 antibodies [11–18].

Glucosamine is a natural component of the glycosaminoglycans found in the cartilage matrix and synovial fluid; when administered exogenously, it affects the cartilage and chondrocytes metabolism, mainly leading to the reverse of the pro-inflammatory and joint-degenerating effects of IL-1 [24, 25]. The prescription-grade crystalline glucosamine sulfate (pCGS) formulation is widely used for the treatment of knee OA thanks to its well-demonstrated effectiveness in improving pain and function [26–31]. Furthermore, pCGS and CS are recommended as chronic background therapy by European Society for Clinical and Economic Aspects of Osteoporosis, Osteoarthritis and Musculoskeletal Diseases (ESCEO) algorithm for knee OA [32–34].

No randomized placebo-controlled trials evaluating the possible symptomatic effects of GS in patients with HOA have been performed. Some positive and promising results were derived from our previous 6-months’ retrospective study, demonstrating the efficacy of pCGS (1500 mg/day) added to conventional therapy, on hand pain and functionality [35]. However, to the best of our knowledge, no data are available in EHOA.

For these reasons, we decided to retrospectively evaluate the possible efficacy of pCGS, as an add-on treatment to conventional therapy for HOA, in comparison to conventional therapy alone, in patients with concomitant EHOA and knee OA. The conventional regimen consisted in education and training in ergonomic principles, exercise program for HOA and acetaminophen or oral NSAIDs, used on-demand.

## Patients and methods

### Study design

The current study was an observational cohort study with retrospective review of medical records, conducted at the Center for the diagnosis and the management of Hand Osteoarthritis, Rheumatology Unit of the Azienda Ospedaliera Universitaria Senese (Italy).

In accordance with national regulations on the conduction of observational analysis [36], the local Ethics Committee was notified of the current retrospective observational study.

According to our routinary care, all patients were informed that their demographical and medical data could be used in a scientific study and provided their written consent for the collection and publication of anonymous data.

The study was registered on http://www.clinicaltrials.gov with number NCT05237596.

### Participants

We analyzed the records, collected since October 2016 to October 2021 in the departmental archives, of all the outpatients affected by concomitant mono-or bilateral knee OA, diagnosed according to the American College of Rheumatology (ACR) criteria [37], and EHOA, defined as the presence of the classical central erosion in at least two IP joints [3, 38], and who have been treated with prescription-grade crystalline glucosamine sulfate (Dona®), in addition to the conventional therapy for HOA or with conventional therapy alone.

We included in our analysis the records of patients of both sexes, aged between 48 and 87 years who had clinical symptoms of HOA for at least 3 months, defined as global hand pain score in the previous 48 h superior to 40 mm on a 0–100 Visual Analogue Scale (VAS) and a functional index for hand osteoarthritis (FIHOA) score of at least 6. Furthermore, to be included in our analysis, patients have had plain radiography of both hands performed within the past 6 months before the first visit at our clinic. Radiographic disease severity was determined based on the Kellgren/Lawrence scoring system and was performed by an expert rheumatologist (A.F.). All individual distal interphalangeal (DIP), proximal interphalangeal (PIP) and carpometacarpal (CMC) joints were scored according to Kellgren and Lawrence grading [39]. The final score was determined by the joint with the highest grade [16].

Patients with a history of any inflammatory joint disease, septic arthritis, previous articular fracture of the concerned joints, or the presence of any other rheumatic diseases that could cause secondary OA, such as hemochromatosis, represented exclusion criteria. Further, patients who underwent therapy with SYSADOAs, other than pCGS, steroids by any route of administration and intra-articular injection of any joint with hyaluronic acid during the previous 6 months were excluded. Also patients treated with intra-muscular or intra-venous bisphosphonates in the previous 6 months were not considered. Other obvious exclusion criteria, considering that in none of these conditions, we prescribe pCGS therapy according to our routinary care, were a known history of allergy to pCGS, to any of the other ingredients of this medicine or to shellfish, as glucosamine is produced from shellfish, significant comorbidities, as diabetes or impaired glucose tolerance, severe cardiovascular, liver or kidney diseases, asthma, phenylketonuria, pregnancy and breast-feeding.

### Treatments

The participants were stratified into two groups based on whether or not pCGS treatment was added to the conventional therapy for EHOA. Thus, pCGS-exposed Group included patients treated with pCGS (Dona®, VIATRIS), in sachets of powder for oral solution, at the dose of 1500 mg glucosamine sulfate once daily, for a total period of 6 consecutive months according to the approved indication for knee OA, in addition to conventional therapy for HOA, while pCGS-unexposed Group included patients treated with conventional therapy alone for 6 consecutive months.

The treatment with pCGS is prescribed in our routinary care, as background therapy, in patients with knee OA, according to the algorithm recommended by ESCEO [14–16]. Thus, the decision by the physician to not prescribe pCGS depends exclusively on patients' contraindications or co-morbidities, as reported above (known history of allergy to pCGS, to any of the other ingredients of this medicine or to shellfish, as glucosamine is produced from shellfish, significant comorbidities, as diabetes or impaired glucose tolerance, severe cardiovascular, liver or kidney diseases, asthma, phenylketonuria, pregnancy and breast-feeding). Furthermore, in some cases, patients decided to not take pCGS because they cannot afford the cost of this drug since it is not covered by our National Health Service (NHS), opposite to NSAIDs and acetaminophen.

The conventional treatment for HOA consisted of education and training in ergonomic principles, exercise program for HOA and acetaminophen or oral NSAIDs, used on-demand. Education and information about HOA were provided to all patients through the use of a 11-page educational booklet (Supplementary material 1). Training in ergonomic principles and pacing of activity (formerly known as “joint protection”) were offered to all patients trough two individual face-to-face sessions with a physiotherapist.

Concerning exercise, in our routinary care, we refer to the program, described by Østerås et al. [40, 41] in order to maintain or increase the flexibility of the metacarpal (MCP), DIP and PIP joints, to strengthen the mm. extensors and abductors pollicis and to potentiate grip strength. Four weekly face-to-face sessions with a trained physiotherapist were offered to all patients, and then they were instructed to perform three home sessions for week.

According to the Italian Society for Rheumatology clinical practice guidelines [42], acetaminophen was prescribed up to a maximum of 1000 mg for 3 times/day and oral non-selective or COX-2 selective NSAIDs for a limited period of time, taking into account all patients’ comorbidities, contraindications and special warnings. Particularly, the treatment with NSAIDs includes diclofenac (single daily dose ≤ 150 mg) or piroxicam (single daily dose ≤ 20 mg) or naproxen (maximum 500 mg for 2 times/day) or aceclofenac (maximum 100 mg for 2 times/day) or ibuprofen (maximum 400 mg for 3 times/day) or celecoxib (single daily dose ≤ 200 mg) or etoricoxib (single daily dose ≤ 60 mg).

### Data collection

Data from routine clinical practice of eligible patients were collected by completion of case report forms during each visit. In particular, the following data were extracted from the chart review and aggregated into a Microsoft Excel® spreadsheet database: demographic and anthropometric measures such as age, gender, and body mass index (BMI), clinical data, as patient’s history, comorbidities and related treatments. This information was self-reported by the patients and checked by evaluation of the referred clinical documentation. Data about HOA characteristics, including disease duration, radiological score, pain severity and algo-functional indexes, and features of other frequently affected joints, as knee and hip, as well as QoL tests were also collected.

### Outcomes

The study parameters were collected during the visits at the out-patient clinic specialized for the diagnosis and management of HOA of our institution, which are scheduled every 3 months for each patient. In case of impossibility for the patients to come to the center for follow-up visits, we usually reach them by phone.

In the present study, we considered only data regarding HOA, which is the focus of this paper and we did not report any assessments about knee OA.

The primary outcome criteria were the change from baseline to month 6 in the patient’s assessment of global spontaneous hand pain, perceived in the previous 48 h, by VAS (0–100 mm), with 0 representing the absence of pain and 100 the maximum imaginable pain, and in hand function by FIHOA score.

The FIHOA score represents a quantitative measure of functional disability of the hands; it contains 10 items and is an investigator-administered questionnaire. Patients are asked to answer each item using a four-point Likert scale: 0 = possible without difficulty, 1 = possible with slight difficulty, 2 = possible with considerable difficulty, 3 = impossible. The range of scores is 0–30 and the highest values indicate the worst functionality. The validated Italian version of FIHOA was used [43, 44].

Among secondary outcomes we evaluated the change in the duration of morning stiffness, measured in minutes and based on the self-report of patients. Further secondary endpoints were the Italian version of the Health Assessment Questionnaire (HAQ), the medical outcomes study 36-item short form (SF-36), symptomatic drug consumption from baseline to month 6 of follow-up and the percentage of treatment responders at 3 and 6 months, according to the Outcome Measures in Rheumatology (OMERACT) and Osteoarthritis Research Society International (OARSI) criteria [45–49].

For both primary and secondary outcomes, the target hand was defined as the patient’s most symptomatic hand or, when patients referred both hands as equally painful, their dominant hand. The acetaminophen and NSAIDs consumption was calculated asking the patients at each visit the number of tablets taken weekly.

Furthermore, we evaluated all adverse events, whether reported spontaneously by the patients or observed by the physician, were recorded, describing the severity and any possible relationship with the treatment.

### Statistical analysis

A sample size of at least 50 patients per group was adequate for evidencing a difference between the two groups of at least 10 mm in the change in global hand pain score between baseline and the 6-month end-point, hypothesizing a standard deviation (SD) of 15 mm, with a power of 80% and an alpha error of 0.05.

Continuous variables were evaluated in term of means and standard deviation (SD), after checking their normality. For categorical relative frequencies (%) were reported. Parametric univariate tests (*p*-values were referred to Fisher Exact for frequencies and *t*-test for means) were used for evaluating possible differences between pCGS and controls at baseline.

Mean changes from baseline in the outcomes of interest, i.e., VAS pain, VAS rigidity, FIHOA, HAQ, SF-36 physical component score (PCS) and mental component score (MCS), were compared using a generalized linear model (GLM) with repeated measures, calculating the differences between exposed and unexposed, over time. The same was due to for the weekly consumption of symptomatic drugs.

For all analyses, a *p* value of less than 0.05 was considered as statistically significant, applying the Bonferroni’s correction in the sensitivity analysis.

All analyses were performed using the SPSS 20.0 for Windows (SPSS Inc., Chicago, Illinois).

### Data entry and management

Data were collected through the prespecified case report forms we usually fill during our ambulatory care. Paper-based data were stored in locked cabinets with restricted access to the rheumatologists of the Center for the diagnosis and the management of Hand Osteoarthritis, Rheumatology Unit of the Azienda Ospedaliera Universitaria Senese (Italy). Electronic data were stored in a password-protected database with secured and restricted access. Patients were identified by study ID with other information potentially identifying individuals removed.

## Results

### Participants

**Fig. 1:**
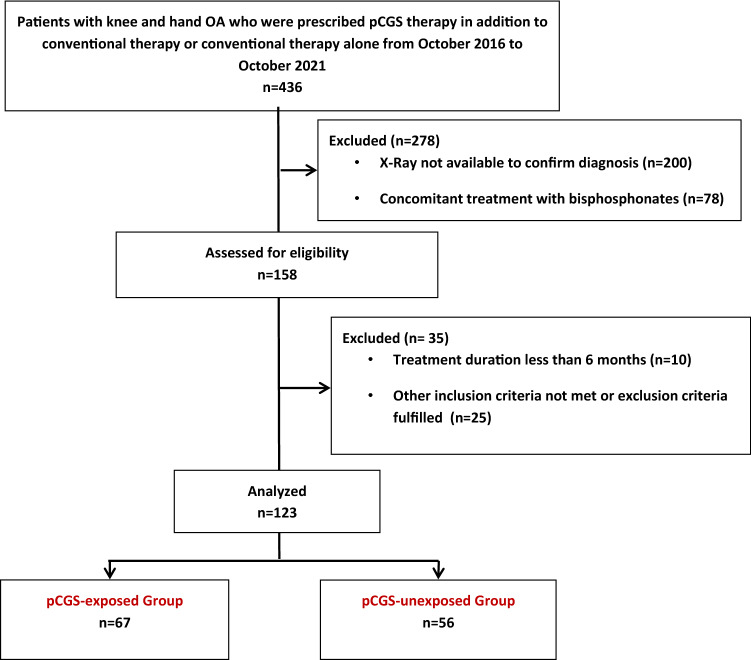
Flow diagram of the study population. *OA* osteoarthritis, *pCGS* prescription-grade crystalline glucosamine sulfate; *DMARDs* disease modifying anti-rheumatic drug

We revised the medical charts of 436 patients with concomitant EHOA and knee OA. Of these, 200 patients were excluded because of the unavailability of recent hand radiographs to confirm the diagnosis of EHOA and 78 for the concomitant treatment with intra-muscular bisphosphonates. Furthermore, we excluded other 35 patients, because they did not meet other inclusion criteria or fulfilled other exclusion criteria. Only 10 patients presented a poor compliance to the combination program prescribed and were not included. Finally, a total of 123 patients with complete data were included in our analysis as follows: 67 in the pCGS-exposed Group and 56 in the pCGS-unexposed Group (Fig. 1).

Baseline demographic and clinical characteristics of the study population are reported in Table 1. The two groups were comparable, except for the percentage of acetaminophen users that was greater in the pCGS-unexposed Group (*p *< 0.05); participants were mainly women with a mean age (SD) of 66.6 (9.6) years and a disease duration of 3.7 (1.7) years.

**Table 1:** Demographic and clinical characteristics of the study population

	pCGS-exposed Group (*n* = 67)	pCGS-unexposed Group (*n* = 56)	*p*-value
Age (years)	66.5 (9.5)	66.8 (9.9)	0.88^a^
Sex males/females, *n*	5/62	3/53	0.63^b^
Education, *n* (%)			
Primary school	16 (24)	13 (23)	0.93^b^
Middle school	18 (27)	18 (32)	0.52^b^
High school	26 (39)	23 (41)	0.52^b^
University	7 (10)	2 (4)	0.14^b^
BMI (kg/m^2^)	25.1 (3.4)	24.0 (3.6)	0.09^a^
Disease duration (years)	3.7 (1.7)	3.7 (1.7)	0.93^c^
Radiographic Score (K/L grade), *n* (%) (°)			
II	26 (39)	21 (37)	0.88^b^
III	41 (61)	35 (63)	0.88^b^
Swollen IP joints			
Right hand	0.9 (1.4)	1.2 (1.9)	0.25^c^
Left hand	0.8 (1.5)	1.2 (1.8)	0.26^c^
Patients with concomitant TMC OA, *n* (%)	56 (83.6)	44 (79)	0.50^b^
Current smokers, *n *(%)	5 (7)	9 (16)	0.13^b^
CV disease, *n* (%)	11 (16)	11 (20)	0.63^b^
Hypertension, *n* (%)	30 (45)	26 (46)	0.86^b^
Autoimmune thyroiditis, *n* (%)	22 (33)	14 (25)	0.43^b^
Hypercholesterolemia *n* (%)	42 (63)	32 (57)	0.53^b^
Hypertriglyceridemia, *n* (%)	8 (12)	2 (4)	0.09^b^
ESR (mm/h)	22.5 (14.0)	18.6 (11.8)	0.05^a^
CRP (mg/dl)	0.24 (0.19)	0.42 (1.33)	0.27^a^
VAS pain (0–100 mm)	66.8 (18.5)	66 (17.5)	0.80^a^
VAS stiffness (min)	10.8 (10)	13.5 (13.8)	0.21^a^
FIHOA (0–30)	10.8 (4.8)	11.1 (4.7)	0.74^a^
HAQ (0–3)	0.75 (0.5)	0.77 (0.5)	0.81^a^
SF-36 PCS	40.6 (11.1)	41.4 (10.2)	0.69^a^
SF-36 MCS	36.7 (16.6)	37.2 (15.9)	0.84^a^
Acetaminophen users, *n* (%)	32 (48)	37 (66)	**0.04**^b^
NSAIDs users, *n* (%)	41 (61)	29 (52)	0.29^b^

Except where indicated otherwise, values are expressed as mean ± SD

The significance of bold value is *p* < 0.05

*pCGS* prescription-grade crystalline glucosamine sulfate, *BMI* body mass index, *K–L grade* Kellgren–Lawrence grade, *IP* interphalangeal, *TMC OA* trapeziometacarpal osteoarthritis, *CV* cardiovascular, *ESR* erythrocyte sedimentation rate, *CRP* C-reactive protein, *VAS* Visual Analogue Scale, *FIHOA* Functional Index for Hand Osteoarthritis, *HAQ* Health Assessment Questionnaire, *SF-36 PCS* medical outcome study 36-item short form physical component summary, *SF-36 MCS* medical outcome study 36-item short form mental component summary, *NSAIDs* non-steroidal anti-inflammatory drugs

°Determined by the joint with the highest grade

^a^Unpaired *t* test

^b^Chi square test

^c^Mann–Whitney test

### Primary and secondary clinical outcomes

**Fig. 2:**
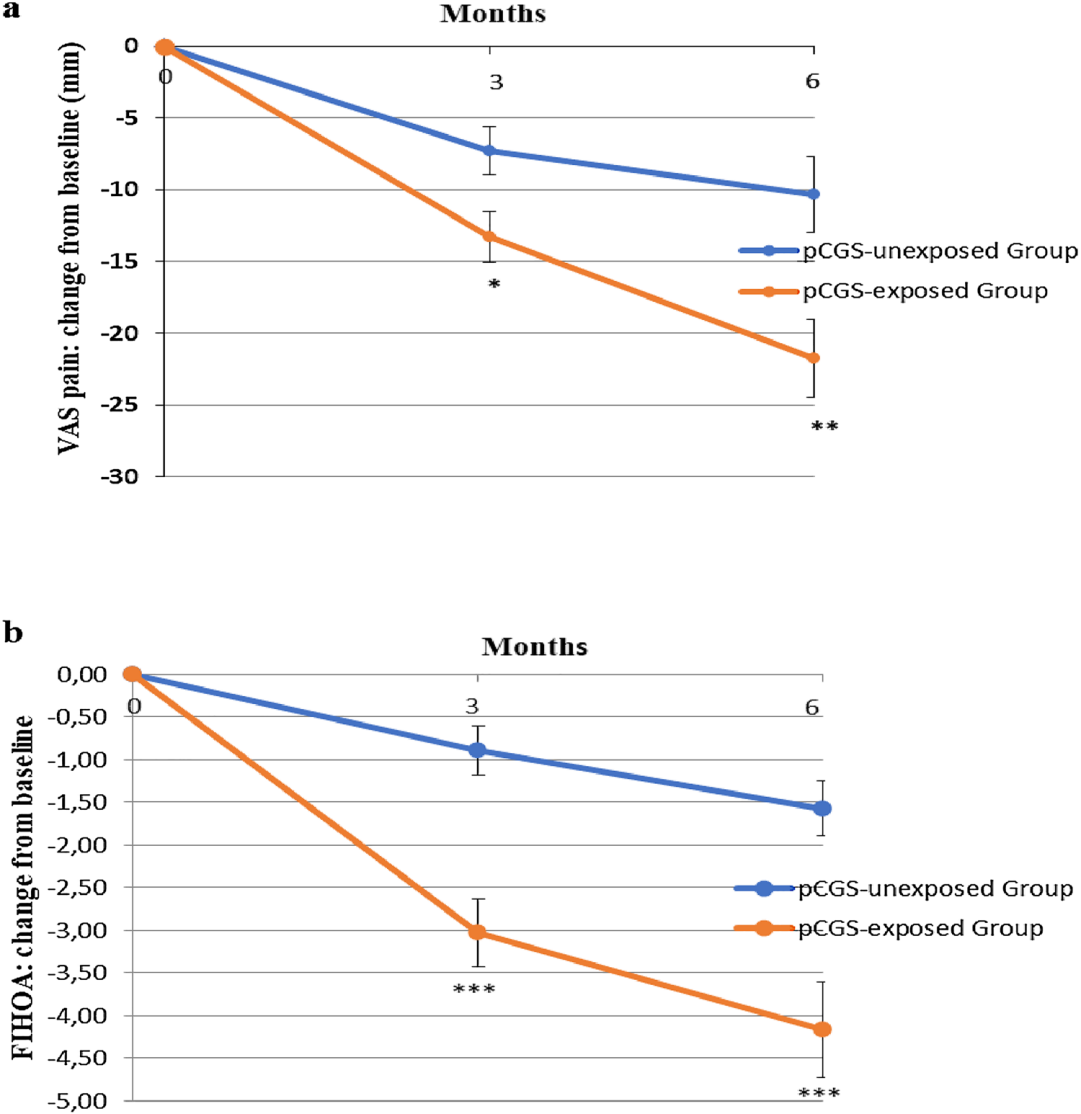
Means with standard error changes in the Visual Analogue Scale (VAS) (0–100 mm) (**a**), and in the Functional Index for Hand Osteoarthritis (FIHOA) score (0–30) (**b**), after 3 and 6 months in prescription-grade Crystalline Glucosamine sulfate (pCGS)-exposed Group and pCGS-unexposed Group. pCGS-exposed Group vs pCGS-unexposed Group: *p < 0.05; **p < 0.01; ***p < 0.001

Figure 2a shows the changes in the patient’s assessment of global hand pain, calculated by VAS (0–100 mm), after 3 and 6 months of treatment. The mean change between baseline and month 6 in VAS pain in pCGS-exposed Group was greater than in pCGS-unexposed Group (mean −21.79 [SD 22.18] versus −10.33 [SD 19.99] mm; mean between-group difference −11.46 [*p* < 0.01]) (Fig. 2a). The difference between groups was slightly significant after 3 months of treatment (*p* < 0.05) and became more evident at 6 months (*p* < 0.01) (Fig. 2a).

The changes in FIHOA scores at 3 and 6 months were reported in Fig. 2b. Similarly, to VAS pain, the mean change from baseline and month 6 in FIHOA was more pronounced in patients treated with pCGS than in unexposed ones (mean −4.16 [SD 4.63] versus −1.57 [SD 2.37]; mean between-group difference −2.59 [*p* < 0.001]). The statistical significance between groups was reached already at 3 months and maintained until 6 months (*p* < 0.001) (Fig. 2b).

**Fig. 3:**
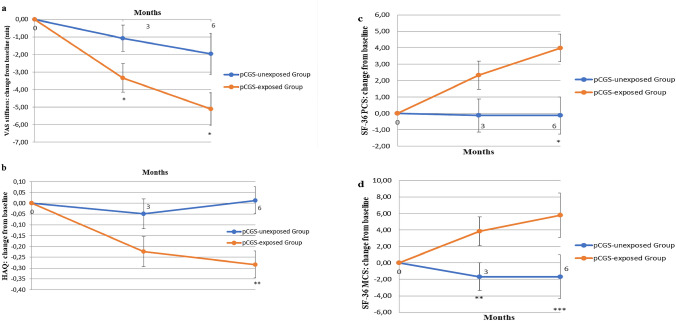
Means with standard error changes in morning stiffness, measured by Visual Analogue Scale (VAS) (minutes) (**a**), in the Health Assessment Questionnaire (HAQ) (0–3) (**b**), in the Physical Component Score (PCS) of the Short-Form Survey 36 (SF-36) (**c**), and in the Mental Component Score (MCS) of SF-36 (**d**), after 3 and 6 months in prescription-grade Crystalline Glucosamine Sulfate (pCGS)-exposed Group and pCGS-unexposed Group. pCGS-exposed Group vs pCGS-unexposed Group: *p < 0.05; **p < 0.001; ***p < 0.001

The results about the secondary outcomes are shown in Fig. 3a–d. The mean change between basal time and the end of follow-up in VAS stiffness was higher in patients treated with pCGS than in unexposed (mean −5.11 [SD 7.54] versus −1.96 [SD 8.70] minutes; mean between-group difference −3.15 [*p* < 0.05]). At six months the mean change from baseline for HAQ was −0.28 [SD 0.59] in pCGS-exposed Group and 0.01 [SD 0.46] in pCGS-unexposed Group with a mean between-group difference of −0.29 (*p* < 0.01). The difference between groups in VAS stiffness became statistically significant at 3 months and remained unchanged at 6 months (*p* < 0.05), while for HAQ the statistical significance between groups was reached at the end of follow-up (Fig. 3a, b). SF-36 significantly improved through the follow-up in the pCGS-exposed Group with a mean change between basal time and the end of follow-up in PCS of 3.98 [SD 9.21] versus −0.12 [SD 6.39] in the pCGS-unexposed Group with a mean between-group difference of 4.1 (*p* < 0.05) (Fig. 3c). Also, the mean change between baseline and month 6 in SF-36 MCS was greater in pCGS-exposed Group compared to pCGS-unexposed Group (mean 5.77 [SD 13.3] versus −1.67 [SD 9.51]; mean between-group difference 7.44 [*p* < 0.001] (Fig. 3d).

In Table 2 we reported the values of primary and secondary outcomes expressed as mean and standard deviation in the two studied groups through the follow-up period.

**Table 2:** Mean ± standard deviation (SD) of primary and secondary outcomes at each evaluation time

Parameter	pCGS-exposed Group (*n* = 67)			pCGS-unexposed Group (*n* = 56)		
	Baseline (T0)	3-months (T1)	6-months (T2)	Baseline (T0)	3-months (T1)	6-months (T2)
VAS pain (mm)	66.8 ± 18.5	53.5 ± 21.31***	45 ± 24.01***	66 ± 17.4	58.7 ± 19.2**	55.7 ± 22.2**
FIHOA score	10.8 ± 4.8	7.8 ± 4.6***	6.7 ± 4.6***	11.1 ± 4.7	10.2 ± 5.1	9.5 ± 5.1*
VAS stiffness (min)	10.8 ± 10	7.5 ± 7.5**	5.7 ± 5.5***	13.5 ± 13.8	12.5 ± 12.8	11.6 ± 11
HAQ	0.7 ± 0.5	0.5 ± 0.4**	0.4 ± 0.3***	0.7 ± 0.5	0.7 ± 0.5	0.7 ± 0.5
SF-36 PCS	40.6 ± 11.1	42.9 ± 10.2	44.7 ± 10.2**	41.4 ± 10.2	41.3 ± 11.1	41.3 ± 11.1
SF-36 MCS	36.7 ± 16.6	40.4 ± 14.4	42.4 ± 14.5**	37.2 ± 15.9	35.5 ± 16.3	35.5 ± 16.3

*pCGS* prescription-grade Crystalline Glucosamine Sulfate, *VAS* Visual Analogue Scale, *FIHOA* Functional Index for Hand Osteoarthritis, *HAQ* Health Assessment Questionnaire, *SF-36 PCS* medical outcome study 36-item short form physical component summary, *SF-36 MCS* medical outcome study 36-item short form mental component summary

* *p*  <  0.05 T2 vs T0; ** *p * <  0.01 T1, T2 vs T0; *** *p*  <  0.001 T1, T2 vs T0

Concerning the use of symptomatic drugs, at baseline there was a significant major consumption of acetaminophen in pCGS-unexposed Group than in pCGS-exposed Group. It remained stable over time in pCGS-unexposed Group, while significantly decreased in the pCGS-exposed Group (*p* < 0.001). The difference between groups became more significant (*p* < 0.001) after 3 and 6 months (Table 3). After adjusting our analyses for the differences between the two groups in the weekly use of acetaminophen at baseline, the results did not change (*p* < 0.0001 for the comparison at 3 and 6 months). Non-selective or COX-2 selective NSAIDs consumption resulted significantly reduced at 3 and 6 months (*p* < 0.001) in the pCGS-exposed Group, while the unexposed patients experienced a slight, but not significant decrease of NSAIDs use during the follow-up. The differences between the groups were significant (*p* < 0.01) at the end of the follow-up (Table 3).

**Table 3:** Symptomatic drugs consumption at each evaluation time

Parameter	pCGS-exposed Group (*n* = 67)			pCGS-unexposed Group (*n* = 56)		
	Baseline (T0)	3-months (T1)	6-months (T2)	Baseline (T0)	3-months (T1)	6-months (T2)
Acetaminophen consumption (*n* of 500 mg-tablets/week)	1.9 (2.7)	1.1 (2.2)°°°	0.9 (1.8)°°°	3.1 (3.7)*	3.2 (3.7)***	3.2 (3.3)***
NSAIDs consumption (*n* of tablets/week)	2.3 (2.3)	1.3 (1.5)°°°	0.6 (1.7)°°°	2.1 (2.5)	1.5 (2.0)	1.4 (2.1)**

Data are reported as mean ± SD

*pCGS* prescription-grade Crystalline Glucosamine Sulfate, *NSAIDs* non-steroidal anti-inflammatory drugs

^*^p < 0.05 pCGS-exposed Group vs pCGS-unexposed Group; **p < 0.01 pCGS-exposed Group vs pCGS-unexposed Group; ***p < 0.001 pCGS-exposed Group vs pCGS-unexposed Group; °°°*p* < 0.001 T1, T2 vs T0

The pCGS-exposed Group had a greater rate of responders, according to OMERACT-OARSI criteria, compared to pCGS-unexposed Group both at 3 (23.8 versus 3.5%, between-group difference = 0.001) and 6 months (50.7 versus 19.6%, between-group difference < 0.001).

### Safety

With regard to the safety analysis, pCGS treatment resulted safe and well tolerated. Only a minority of the patients treated with pCGS (13%) reported side effects, all transient and of light intensity that no one had to interrupt the therapy. The more frequent side effects in this group were diarrhea reported in four patients and dyspepsia/nausea in three patients. Three of the four patients suffering from diarrhea received 1 week therapy with probiotics with resolution of the disorder, while dyspepsia and nausea resolved spontaneously after few days. 21% of the patients in the pCGS-unexposed Group experienced several adverse events, mainly epigastralgia and increase of systemic blood pressure. The five patients suffering of epigastralgia needed a brief course of oral pump inhibitor therapy. Fortunately, the increase of systemic blood disease was only transient and did not need any specific treatment (Table 4).

**Table 4:** Number (%) of patients with treatment-related adverse events in the study population

	pCGS-exposed Group (*n *= 67)	pCGS-unexposed Group (*n* = 56)
Epigastralgia	2 (2.9%)	5 (8.9%)
Dyspepsia/nausea	3 (4.4%)	2 (3.5%)
Diarrhea	4 (5.9%)	1 (1.7%)
Increased blood pressure	0 (0%)	4 (7%)
Total n of patients with adverse event	9 (13.2%)	12 (21.1%)

*pCGS* prescription-grade Crystalline Glucosamine Sulfate

## Discussion

This observational retrospective study demonstrates the ability of the prescription-grade crystalline glucosamine sulfate of improving pain and function, when used in combination to conventional therapy, in patients with erosive osteoarthritis of the hand. The improvement of both parameters was already significant after 3 months of therapy, but became more evident after 6 months. In agreement with previous results, the effects on hand functionality, measured by FIHOA, resulted more pronounced than those on VAS pain [22, 35].

Furthermore, a significant difference in the change of duration of morning stiffness, HAQ and SF-36 was observed between the two studied groups. In our opinion, the significant increase of PCS and MCS of SF-36 at 6 months (*p* < 0.001) is noteworthy, considering that EHOA is known to be associated with a high impact on quality of life [9, 50, 51].

In line with the above-reported beneficial effects, the treatment with pCGS resulted in a relevant decrease in symptomatic drugs intake. Particularly, we observed a significant reduction in acetaminophen and selective or non-selective NSAIDs use, after 3 and 6 months, with a significant difference between the studied groups. These findings suggest an overall better control of the disease symptoms with pCGS therapy in agreement with previous studies [52, 53]. In particular, the Pharmaco-Epidemiology of GonArtrhoSis (PEGASus) study reported that, among the analyzed SYSADOAs (pCGS, glucosamine hydrochloride, pCS, avocado soybean unsaponifiables and diacerein), only pCGS was able to induce a reduction of 36% of NSAIDs intake (OR 0.64; 95% CI 0.45–0.92) [53].

Furthermore, in the present study, we observed a lower amount of side effects, especially of the gastric ones, in the pCGS Group, probably due to the overall decrease of symptomatic drugs consumption.

To the best of our knowledge, this is the first study evaluating the effectiveness of pCGS, as an add-on therapy to conventional treatments in patients with EHOA, a very complex disease which is actually lacking of effective therapies. It is still debated if EHOA represents a more advanced phase of the classical HOA or rather a separate and more severe entity; its pathogenetic mechanisms are still poorly known [7, 8]. For decades, an important role in the underlying processes of EHOA was attributed to inflammation, but the disappointing and contrasting results derived from trials evaluating the treatment with the classical and biological anti-rheumatic drugs in this pathology induces an important reflection about the need for a better understanding of the basic disease mechanisms and the potential therapeutic targets [5]. Indeed, several clinical trials assessing the efficacy of anti-TNF agents and other anti-cytokine inhibitors in EHOA failed to reach the primary outcomes. Particularly, no differences in erosive progression were reported after 12 months between patients treated with adalimumab 40 mg subcutaneously every 2 weeks or placebo in a double-blind RCT [11]. In addition, other two double-blind RCTs failed to demonstrate significant improvement of pain and other outcomes after adalimumab therapy versus placebo [12, 13]. Also, etanercept resulted not superior to placebo in reducing VAS pain after 24 weeks in a 1-year double-blind RCT [15]. Conversely, in a single-blind study, monthly intra-articular injections of infliximab into the affected joints led to a significant improvement of pain after 12 months compared to intra-articular injections of saline [54]. Also, the data about targeting IL-1 and IL-6 are not very encouraging. Indeed, a recent placebo-controlled randomized study did not show significant improvement of pain after 24 weeks of therapy with lutikizumab 200 mg subcutaneously every 2 weeks [17]; similarly, two infusions, 4 weeks apart, of tocilizumab resulted no more effective than placebo in inducing pain relief at week 4, 6, 8 or 12 [18]. In agreement with these clinical trials on biologics, a recent study on HOA patients showed poorly detectable IL-1β concentrations and minimal inflammasome activity in the peripheral blood mononuclear cells of both erosive and non-erosive HOA patients [55]. Furthermore, the results derived from trials assessing the efficacy of the classical DMARDs, as hydroxychloroquine, methotrexate and colchicine, were not more positive [14, 16, 19, 20, 56]. Conversely, intravenous and intra-muscular clodronate, a first-generation bisphosphonate, currently registered in Europe for the treatment of postmenopausal osteoporosis, was demonstrated as effective in reducing pain and disability in patients with EHOA [57, 58].

Altogether, these data might question the pivotal role of pro-inflammatory cytokines in the complex pathogenetic processes involved in HOA and suggest that the treatments targeting a single mechanism of action may be insufficient [59].

It is likely that the positive results obtained in the current study can beneficiate of a multimodal approach rather than a single pharmacological agent. According to EULAR guidelines for HOA [21], our study population was treated with a combination of non-pharmacological (education, training in ergonomic principles and exercise) and pharmacological interventions (acetaminophen or NSAIDs on-demand) and some patients received also pCGS for the concomitant knee OA, as chronic background therapy [32–34]. The compliance to this multimodal therapeutic program was good; this was probably due to the education about the nature of the disease and the therapeutic objectives, offered to all patients. Indeed, it is recognized that these measures have minimal effect on OA symptoms, but they are essential for treatment adherence [32].

Glucosamine is a natural compound of glycosaminoglycans in the cartilage matrix and synovial fluid. It is commercially available as glucosamine hydrochloride, derived from an extraction process and often used as a nutraceutical or over-the-counter (OTC) product or glucosamine sulfate, a more complex compound, obtained only by a proprietary semi-synthetic route and stabilization process, responsible for the production of the pCGS [60–63]. pCGS, administered as once-daily dose of 1500 mg, is the only glucosamine formulation highly bioavailable and able to reach therapeutic concentrations at the site of action [64–67].

pCGS is widely used for the treatment of knee OA for its symptomatic and disease-modifying effects, explained through several different mechanisms. Pre-clinical studies demonstrated the ability of pCGS to inhibit superoxide-radical production, inducible nitric oxide synthesis, the COX-2 and prostaglandin E2 (PGE2) generation, probably responsible for the relatively fast onset of symptomatic action demonstrated in previous clinical trials. Furthermore, in vitro studies on OA chondrocyte cultures demonstrated that the above-described mechanism of action is mediated by the inhibition of the nuclear factor kappa B (NF-kB) pathway, activated by IL-1 during the inflammatory process [24, 25, 66].

The effectiveness of pCGS has been well demonstrated in knee OA in three high-quality trials which showed a mild–moderate effect on WOMAC pain ( effect size of 0.27) and, WOMAC function subscales (effect size 0.33) and on Lequesne algo-functional index [26, 27, 29]. A network meta-analysis by Gregori et al. [68], who analyzed published long-term (12 months) RCTs in knee OA, demonstrated that, when studies at high risk of bias were excluded, pCGS was the only analyzed drug able to induce a significant improvement of pain and physical function. Similar results were reported in a recent systematic review and network meta-analysis with a 6-month time horizon [69].

Unfortunately, the treatment with pCGS in patients with HOA has never been evaluated in RCTs; for this reason, this drug is not included in the update of the 2018 EULAR recommendations for the management of HOA. In a previous retrospective study, for the first time, we evaluated the effects of pCGS in addition to conventional therapy compared to usual therapy alone in 108 patients affected by non-erosive HOA. The treatment with pCGS resulted associated to a more significant improvement of pain and function, as well as to a reduction of concomitant symptomatic drugs intake [35]. The present study shows similar results in erosive osteoarthritis of the hand, suggesting a promising and potential role of pCGS as add-on therapy to conventional treatment in this pathologic condition.

The main limitation of the present analysis consists in its observational retrospective design with all the limitations inherent to a not randomized, not blinded and not placebo-controlled trial. In particular, the lack of a placebo arm could have led to overestimate the positive results in pCGS Group, considering that HOA patients are very susceptible to placebo effects [70, 71]. In addition, the small sample size did not allow us to perform any sub-group analysis or to adjust our analysis according to confounder factors, such as comorbidities. Then, demographic and clinical characteristics of the two studied groups were comparable at baseline, except for the percentage of acetaminophen users which was higher in the pCGS-unexposed Group. Thus, although this difference was only minor, it could have affected our results. Furthermore, although we selected as primary outcomes VAS pain and FIHOA score, according to the Osteoarthritis Research Society International (OARSI) and ESCEO recommendations for the conduct of pharmacological clinical trials in HOA [72, 73], we are aware that FIHOA includes some items culturally challenging or outdated and thus, it was rejected as a core outcome measure for HOA trials [74]. Other limitations were represented by the duration of 6 months of the follow-up and by the lack of any standardization for NSAIDs consumption. Further, this study was performed in a single center and included only EHOA with certain inclusion criteria (e.g. VAS pain > 40 mm and FIHOA > 6), so the results may not be generalisable to every setting.

In conclusion, this observational retrospective study showed that oral pCGS, at the dosage of 1500 mg once daily, used as an add-on therapy to conventional treatments was more effective than usual care alone in improving hand pain and functionality in patients with EHOA. This symptomatic effect was evident already after 3 months of therapy and persisted until 6 months. These findings can be promising; however, they should be considered with caution, and confirmed in future RCTs.

## Supplementary Information

Below is the link to the electronic supplementary material.

Supplementary file1 (PDF 370 KB)
