# Supplementary material for: Prescription-grade crystalline glucosamine sulfate as an add-on therapy to conventional treatments in erosive osteoarthritis of the hand: results from a 6-month observational retrospective study
Source: Aging Clin Exp Res. 2022 May 30;34(7):1613–25. doi: 10.1007/s40520-022-02151-7 (PMC9246990; doi:10.1007/s40520-022-02151-7)
Supplement: Supplementary file 1 — Supplementary file1 (PDF 370 KB) [file 40520_2022_2151_MOESM1_ESM.pdf]

Azienda ospedaliero-universitaria Senese

Dipartimento di Medicina interna e specialistica
Ambulatorio Centro di diagnosi e trattamento dell'artrosi della mano
Responsabile dott.ssa Antonella Fioravanti

U.O.C. Reumatologia
Direttore professor Bruno Frediani

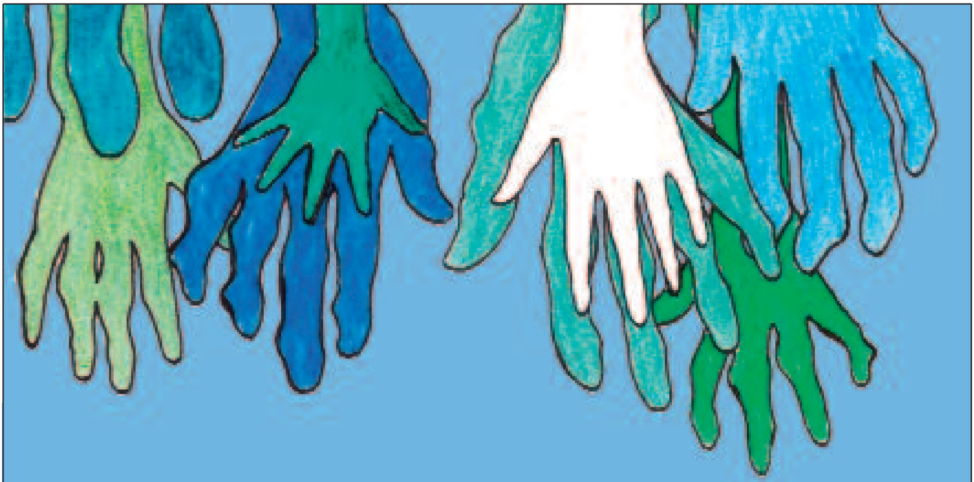

Dieci domande per conoscere, curare e prevenire l'artrosi della mano

A cura di
Antonella Fioravanti, Sara Tenti, Fabiola Rossi

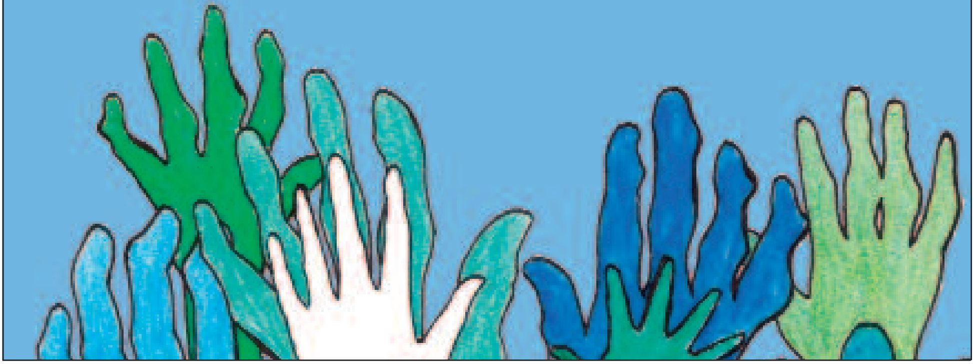

Centro di diagnosi e trattamento dell'artrosi della mano

Ambulatorio 37, terzo lotto, piano 1S
Dall'ingresso centrale dell'ospedale seguire la striscia azzurra

Responsabile

Dott.ssa Antonella Fioravanti
E-mail: antonella.fioravanti@ao-siena.toscana.it

Collaboratrice

Dott.ssa Sara Tenti

Le visite ambulatoriali vengono effettuate il giovedì pomeriggio.

Centro unico di prenotazione visite (Cup)

Orario telefonico: lunedì - venerdì 8.00-18.00; sabato 8.30-13.00
Tel. 0577 767676
Orario di sportello: lunedì - venerdì 7.45-18.15; sabato 7.45-13.00

Tutte le informazioni relative all'Azienda ospedaliero-universitaria Senese sono disponibili nel sito www.ao-siena.toscana.it.

Che cos'è l'artrosi?

L'artrosi, o meglio l'osteoartrosi, è una malattia articolare cronica che colpisce inizialmente la cartilagine articolare e successivamente le altre strutture dell'articolazione, quali la membrana sinoviale, la capsula e i tessuti molli circostanti.
Durante il processo artrosico la cartilagine va incontro a degenerazione e perde la sua funzione ammortizzante, provocando alterazioni nell'osso sottostante, come la formazione di cisti e di osteofiti.
L'artrosi, che pur presenta caratteri prevalentemente degenerativi, spesso si accompagna a segni più o meno intensi di infiammazione.

Quanto è diffusa?

L'artrosi è la malattia reumatica più frequente. Interessa infatti circa il 20% della popolazione e rappresenta nei paesi sviluppati la causa principale di invalidità cronica. Solo in Italia 4 milioni di persone, in particolare anziani (l'80% nei pazienti ha più di 75 anni di età), sono portatrici di tale affezione. L'artrosi continua a colpire un numero sempre maggiore di individui, anche in relazione all'aumento dell'età media della popolazione.

Quali fattori possono causarla?

Nonostante la mole di studi scientifici sulla materia, non sono state ancora individuate con certezza le cause di questa malattia, sebbene siano stati individuati alcuni presunti fattori di rischio.
Tra questi, i più importanti sono:

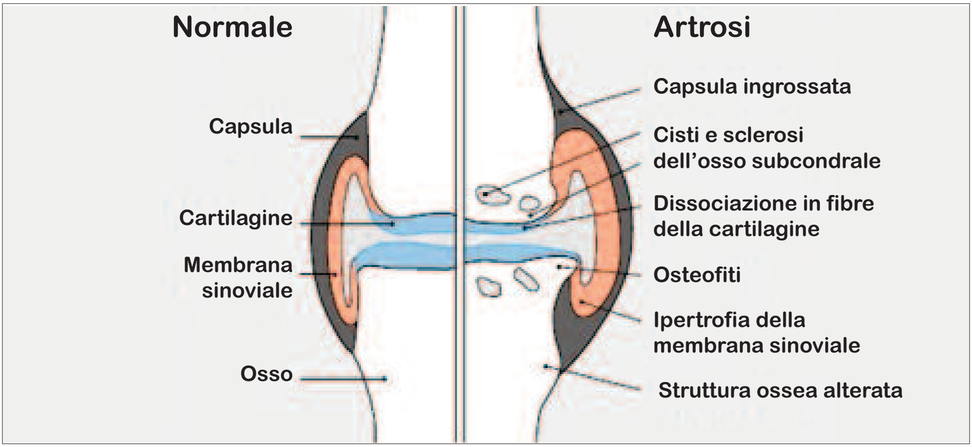

Modificazioni strutturali dell'articolazione artrosica: confronto tra la struttura articolare normale e artrosica

• **Età,** dai 50 anni in avanti. L'artrosi non è un evento fisiologico legato all'invecchiamento, ma il progressivo avanzamento dell'età, associato ad altre cause rappresenta un importante fattore di rischio.

• **Sesso femminile.** La menopausa rappresenta un consistente fattore di rischio. Si ipotizza che gli estrogeni giochino un ruolo non indifferente nell'insorgenza e nella progressione della patologia.

• **Fattori ereditari.** L'importanza del fattore genetico è particolarmente evidente nell'osteoartrosi della mano, soprattutto nelle forme nodulari, anche se allo stato attuale non sono emerse anomalie genetiche che possano avere incidenza a livello clinico.

• **Etnia**

• **Sovrappeso e obesità.** La comparsa della malattia può essere favorita dall'eccessivo carico articolare e dal moderato stato infiammatorio sistemico proprio delle persone obese. In questo secondo caso, le sostanze liberate dal tessuto adiposo bianco (adipocitochine) agiscono sia sul metabolismo osseo-cartilagineo che sulla regolazione del processo infiammatorio. Tale azione può spiegare il legame esistente tra l'obesità e l'artrosi a carico delle articolazioni non portanti come la mano.

• **Alterazioni metaboliche**, quali il diabete mellito, la gotta, l'ocronosi, l'emocromatosi e la malattia di Wilson.

• **Malformazioni**

• **Attività lavorative o sportive intense o incongrue**, fattore indirettamente testimoniato dell'assenza di osteoartrosi nell'arto sede di emiparesi e, al contrario, evidenziato dall'associazione con particolari attività.
Ne risultano infatti vittime:
- pianisti (artrosi del IV e V dito della mano destra)
- pugili e sarti (rizoartrosi)
- giocatori di pallavolo o di baseball (noduli di Heberden)
- raccoglitori di cotone (Missouri Metacarpal Syndrome e osteoartrosi della II e III metacarpofalangea).

La conoscenza di questi fattori di rischio è fondamentale per la prevenzione della malattia e delle sue riacutizzazioni.

Come si manifesta l'artrosi della mano?

L'artrosi della mano è considerata la tipica espressione dell'artrosi primaria. Può infatti interessare numerose sedi articolari (le artico-

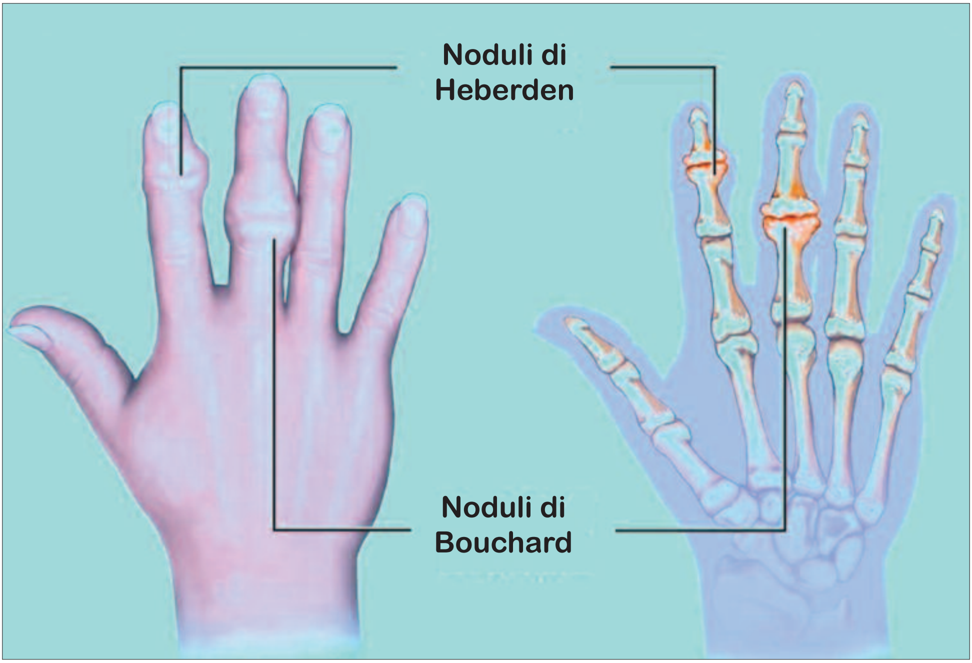

Le deformità articolari tipiche del processo artrosico

lazioni interfalangee prossimali e distali, l'articolazione trapezio-metacarpale e, anche se più raramente, le articolazioni metacarpo-falangee).

Aspetti clinici:

• Dolore meccanico, che aumenta con il movimento e diminuisce con il riposo. Di solito colpisce le articolazioni, in modo intermittente. E' più forte nelle ore serali che in quelle mattutine.

• Dolorabilità alla palpazione e alla mobilizzazione.

• Rigidità articolare (durata inferiore a 30 minuti).

• Limitazione funzionale: legata alla sintomatologia dolorosa nella fase iniziale e alle deformità in quella evoluta.

• Deformità articolari, come per esempio la deviazione laterale delle articolazioni interfalangee prossimali, la sublussazione e l'adduzione della base del pollice.

• Noduli di Heberden e di Bouchard. Soprattutto i primi presentano una consistenza dura, quasi ossea. La cute che li riveste è generalmente normale tranne nelle fasi infiammatorie in cui presenta dolore, tumefazione e calore.

• Rizoartrosi, che conferisce alla mano il tipico aspetto "quadrato". E' spesso di origine secondaria, legata a microtraumatismi cronici e sovraccarico funzionale. Evolve spesso nell'anchilosi in adduzione e flessione del pollice.

Come si riconosce?

La raccolta accurata dei sintomi riferiti dal paziente e l'esame clinico della mano sono importanti per una diagnosi corretta. Al momento attuale non esistono esami di laboratorio specifici per la diagnosi dell'artrosi; la radiografia tradizionale è l'esame strumentale più utilizzato per mettere in evidenza le alterazioni caratteristiche provocate dalla malattia: irregolarità e diminuzione della rima (spazio) articolare, presenza di modificazioni strutturali dell'osso sottostante la cartilagine ed escrescenze ossee ai margini dell'articolazione (osteofiti).

L'ecografia articolare, un esame indolore e facilmente ripetibile, consente di studiare in modo rapido e utile l'intera struttura articolare, compresi tendini, legamenti, membrana sinoviale e cartilagine. Soltanto in casi particolari si deve ricorrere a un approfondimento diagnostico tramite la tomografia computerizzata (TC) o la risonanza magnetica nucleare (RMN).

Quali sono le terapie utilizzabili?

Il trattamento dell'artrosi della mano prevede un approccio multidisciplinare, seguendo uno schema terapeutico strutturato in base alle esigenze del paziente. In linea generale la terapia dell'artrosi prevede:

• Educazione del paziente

E' una componente fondamentale nel trattamento dell'artrosi. Una volta effettuata la diagnosi, il medico informa il malato sulla natura benigna della malattia, gli scopi generali del trattamento e i risultati conseguibili con un'opportuna terapia che non deve limitarsi alla cura del dolore ma mirare ad arrestare o rallentare l'evoluzione e gli esiti della malattia. Trattandosi di programmi terapeutici a lungo termine, è necessario che il paziente comprenda l'importanza di osservare scrupolosamente le diverse misure preventive e terapeutiche prescritte e sia consapevole dei limiti della terapia.

• Norme da seguire:

- Utilizzare strumenti tecnicamente modificati (utensili a pinza, schiaccianoci da tavolo, apribarattoli) per

impedire l'ulteriore usura delle cartilagini interessate dalla malattia.

- Svolgere correttamente le azioni quotidiane (economia articolare), minimizzando gli sforzi e l'affaticamento delle articolazioni ed evitando che una gestualità scorretta o forzata possa aggravare la disabilità. L'obiettivo è evitare lo "stress "articolare". Nello spostamento dei pesi è importante utilizzare leve e impugnature che permettano di non affaticare la presa della mano. Ergoterapia ed economia articolare sono pertanto strettamente connesse.

- Utilizzare i tutori. I tutori si possono distinguere tra quelli "da riposo", di cui avvalersi per lo più durante la notte e nelle fasi infiammatorie della malattia, quelli "funzionali", da utilizzare durante le attività per ridurre le sollecitazioni patologiche e quelli "da correzione", necessari per limitare le deformazioni.

- Effettuare gli esercizi terapeutici prescritti. L'esercizio fisico o chinesiterapia rinforza le masse muscolari e tiene le articolazioni in esercizio, riducendo così la limitazione funzionale e impedendo gli esiti invalidanti. Gli esercizi devono essere attuati in maniera progressiva e graduale senza provocare dolore.

• Terapie non farmacologiche

- Paraffinoterapia. La paraffina, un derivato del petrolio composto da idrocarburi, si trova allo stato solido a temperatura ambiente e fonde intorno ai 50°. Può essere applicata seguendo diversi metodi: pennellature, schiuma, bagno e guanto. Quest'ultima è la tecnica più utilizzata e consiste nello sciogliere il panetto a bagnomaria, inserirvi la mano tre o quattro volte e poi rimuovere l'impacco dopo 20 minuti circa. La paraffina cede il calore ai tessuti sottostanti svolgendo un'azione di termoterapia esogena. L'aumento di temperatura cutanea provoca inoltre un'abbondante sudorazione che accresce la traspirazione cutanea ed elimina le tossine locali.

- Le terapie termali (bagni, fanghi, grotte e docce) rappresentano un valido ausilio alle più moderne terapie mediche. Riducono infatti la sintomatologia dolorosa, le contratture muscolari e l'edema dei tessuti periarticolari migliorando le prestazioni funzionali articolari. L'efficacia della terapia termale si può avvertire fino a 6-9 mesi dalla sua conclusione. La prescrizione medica deve comunque tenere conto delle condizioni generali e dell'età del paziente, della fase di attività e dello stadio della malat-

tia e soprattutto delle eventuali controindicazioni.

• **Terapie farmacologiche sintomatiche**, che devono essere prescritte dal medico curante o dallo specialista.
Risultano necessarie non solo per alleviare il dolore, ma anche per combattere la conseguente impotenza funzionale. È necessario che il paziente segua scrupolosamente i consigli del medico evitando un trattamento "fai da te". I farmaci sintomatici possono essere somministrati per bocca (paracetamolo e altri analgesici, fans e cortisonici) o tramite l'applicazione di cerotti e pomate (fans e capsaicina).

- **Terapie farmacologiche di fondo**: sostanze, anche dette condroprotettori, in grado di ridurre o arrestare l'evoluzione del processo degenerativo articolare.

- **Terapia chirurgico-ortopedica**, indicata in caso di:
1. Dolore refrattario alle terapie mediche generali e locali
2. Deformazioni articolari tali da limitare considerevolmente la funzione articolare.
Sono possibili numerosi tipi di interventi chirurgici, dai più classici, quali la fusione di articolazioni danneggiate (artrodesi), ai più moderni, come artroplastiche o posizionamenti di protesi di ultima generazione. La terapia chirurgica deve essere sempre seguita da una riabilitazione post-operatoria, i cui obiettivi sono da un lato quello di riottenere la funzionalità articolare con un buon recupero del tono muscolare, dall'altro permettere un rapido ritorno del paziente alle comuni attività della vita quotidiana.

- **Terapie fisiche** (elettroterapia, magnetoterapia, ultrasuonoterapia e laserterapia), dotate di un effetto sintomatico ed efficaci nel ridurre la sensazione di dolore e la contrattura muscolare. In linea generale possono essere impiegate come ausilio al trattamento farmacologico ma non sono utilizzabili nei casi di evidenti segni di infiammazione acuta articolare.

È giusto ricorrere alla terapia infiltrativa?

L'infiltrazione consente di somministrare il farmaco direttamente all'interno dell'articolazione colpita, potenziandone l'effetto terapeutico (per aumento della concentrazione) e riducendo gli effetti indesiderati. Possono essere utilizzati cortisonici a lunga durata

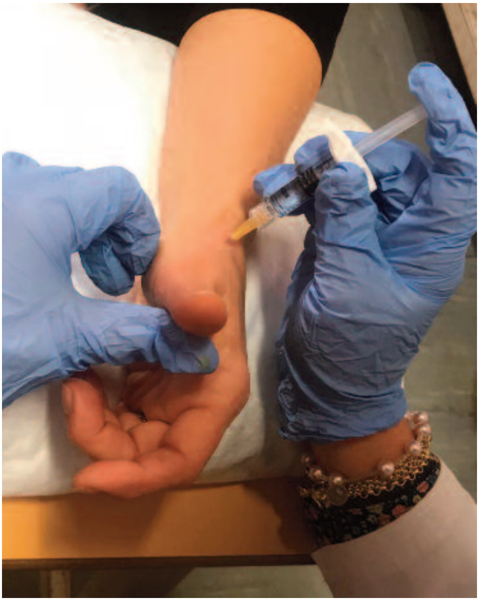

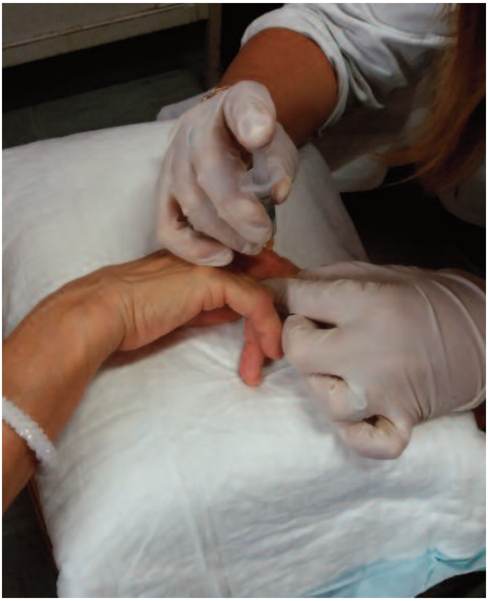

1) Terapia infiltrativa con acido ialuronico dell'articolazione trapezio-metacarpale in una paziente affetta da rizoartrosi; 2) Infiltrazione steroidea della III articolazione interfalangea prossimale in paziente con osteoartrosi erosiva della mano

d'azione, indicati per le fasi infiammatorie e dolorose, e l'acido ialuronico, uno dei componenti fondamentali dei tessuti connettivi dell'uomo. La sua somministrazione intra-articolare riduce il dolore e consente il recupero della funzionalità articolare, consentendo un ripristino delle condizioni normali dell'articolazione e rallentando anche il progresso della malattia.
Ovviamente, essendo terapie invasive che richiedono un adeguato grado di preparazione, devono essere effettuate da specialisti esperti secondo rigorose regole di asepsi.

Artrite e artrosi quali differenze?

Di frequente l'artrosi viene confusa con l'artrite, in particolare con l'artrite reumatoide. L'equivoco è generato dal fatto che entrambe le malattie colpiscono frequentemente le piccole articolazioni della mano e manifestano i primi sintomi con un dolore simile, accompagnato da rigidità e limitazione nei movimenti. Si tratta però di due patologie ben distinte. Innanzitutto, per l'età dei soggetti colpiti: l'artrite è una patologia infiammatoria cronica di origine autoimmune che può svilupparsi in

soggetti di ogni età, anche nei bambini, mentre l'artrosi è una malattia degenerativa che insorge soprattutto dopo i 50 anni.
L'artrite, inoltre, si manifesta con i segni dell'infiammazione articolare quali gonfiore, tumefazione, arrossamento, rigidità, calore e dolori che comportano anche la perdita della capacità motoria delle articolazioni interessate. Le forme più acute possono deformare gravemente le articolazioni e, anche quelle lievi, se non riconosciute e curate adeguatamente, con il passare degli anni, possono acuirsi e provocare gravi problemi alle persone affette.

Come si previene l'artrosi?

Alcuni semplici accorgimenti quotidiani possono contribuire alla prevenzione dell'artrosi oppure - in caso di malattia già avanzata - a limitare l'evoluzione del danno articolare e a ridurre la sintomatologia dolorosa.
Come già visto, l'obesità rappresenta un fattore predisponente e aggravante di molte forme di artrosi. Una graduale riduzione del peso corporeo, in rapporto all'età, alle eventuali patologie associate e alle condizioni generali del soggetto, è un elemento indispensabile per non vanificare gli effetti delle altre terapie.
La frequente associazione dell'artrosi con alcune malattie dismetaboliche (diabete mellito, iperlipidemia, iperuricemia) fa supporre l'esistenza di un nesso tra fattori metabolici e insorgenza della malattia. È pertanto opportuno accertare l'eventuale presenza di tali disturbi in ogni paziente artrosico e intervenire di conseguenza.
Le posizioni posturali e i movimenti scorretti ripetuti più volte quotidianamente comportano una continua e abnorme sollecitazione articolare possono favorire i processi degenerativi e accentuare la sintomatologia dolorosa. La valutazione e l'eventuale correzione delle posizioni posturali normalmente assunte dal paziente sia durante il riposo che durante le ore di lavoro e di tempo libero rappresentano pertanto un momento integrante nel processo di trattamento dell'artrosi.

A chi rivolgersi?

Una prima valutazione compete al medico di medicina generale che, se lo ritiene opportuno, invia il paziente alla valutazione dello specialista reumatologo. Questi ha il

compito di escludere altre forme di coinvolgimento articolare e di impostare la terapia ottimale.
Nei casi più avanzati e refrattari alle terapie convenzionali, può essere utile anche il parere del chirurgo ortopedico, mentre un ruolo di supporto, ugualmente importante, viene svolto dal fisioterapista della riabilitazione.

Depliant realizzato dall'Ufficio Stampa, Redazione e Comunicazione Web
in collaborazione con la UOC Reumatologia
Gennaio 2022
